# Modellierte Individualentwicklung. Humanembryologische Praktiken an der Universität Göttingen in der zweiten Hälfte des 20. Jahrhunderts

**DOI:** 10.1007/s00048-020-00275-3

**Published:** 2020-10-06

**Authors:** Michael Markert

**Affiliations:** Kunstgeschichtliches Seminar und Kunstsammlung, Professur für Materialität des Wissens, Georg-August-Universität Göttingen, Friedländer Weg 2, 37085 Göttingen, Deutschland

**Keywords:** Humanembryologie, Forschungsmodell, Hochschullehre, Praktiken, Materielle Kultur, Human embryology, Research model, Academic teaching, Practices, Material culture

## Abstract

Die Humanembryologische Dokumentationssammlung Blechschmidt, entstanden an der Universität Göttingen im Zeitraum von 1942 bis etwa 1970, stellt eine einmalige Kombination histologischer Schnittserien menschlicher Embryonen und darauf aufbauender, großformatiger und öffentlich zugänglicher Modelle dar. Nicht nur erfolgte diese Sammlungsgründung für die Disziplin der Humanembryologie erstaunlich spät, sondern ist auch in einer zweiten Hinsicht bemerkenswert: Während an anderen Standorten Modelle primär als Forschungsobjekte verstanden wurden und mit einem Lehreinsatz eine Umnutzung stattfand, war für den Göttinger Embryologen Erich Blechschmidt (1904–1992) von Beginn an ein pädagogischer Impetus maßgeblich. Im Beitrag werden die eigenwilligen Merkmale der Blechschmidt’schen Unternehmung vor ihrem disziplinären Hintergrund herausgearbeitet. Der Schwerpunkt liegt dabei auf den beiden für die Humanembryologie zentralen Praktiken des Sammelns und Modellierens und den dadurch in Göttingen geschaffenen Objektbeständen. Diese waren schon im Entstehungsprozess von einem besonderen Spannungsverhältnis von Individualität und Universalität geprägt, das sich auch in der späteren Nutzung der Sammlung spiegelt. Diese führt durch eine spezifische Individualisierung zuvor anonym gemachter Präparate weit aus der ursprünglichen fachwissenschaftlich-anatomischen Bestimmung in die breite gesellschaftliche Debatte um den ethischen Status menschlicher Embryonen und den Schwangerschaftsabbruch.

## Abstract (en)

The Human Embryology Collection at the Centre of Anatomy Göttingen, created between 1942 and 1970, represents a unique interrelation of histological sectional series of human embryos and large-format physical models open to the public based on them. The collection was established long after the heyday of human embryology. It is also remarkable in another aspect: while usually models within the discipline are considered research objects, Göttingen embryologist Erich Blechschmidt (1904–1992) based his understanding on a pedagogical impetus. The article highlights the distinctive and unconventional features of Blechschmidt’s undertaking against its disciplinary background. My focus lies on the two practices that are central to human embryology—collecting and modelling—, as well as the derived collection stocks. The special tension between individuality and universality that already characterized the process of their creation is also reflected in the later use of the collection. This tension allowed Blechschmidt to utilize the models in embryological research and anatomical teaching as well as in the broad social debate on abortion and the ethical status of human embryos.

Im Untergeschoss des Zentrum Anatomie der Universitätsmedizin Göttingen werden in einer öffentlich zugänglichen Schausammlung auf 200 Quadratmeter Ausstellungsfläche Dutzende großformatige Kunststoffmodelle menschlicher Embryonen präsentiert (Abb. 1). Verkörpert findet sich in diesem Teil der „Humanembryologischen Dokumentationssammlung Blechschmidt“ (im Folgenden kurz Blechschmidt-Sammlung) die morphologisch-anatomische Embryonalentwicklung des Menschen vom Zweizellstadium bis zum Ende der Embryonalphase nach acht Entwicklungswochen. Die Referenz der extrem detaillierten Modelle in vielhundertfacher Vergrößerung sind histologische Schnittserien menschlicher Embryonen, die für Besucher*innen unsichtbar im selben Gebäude verwahrt werden und den zweiten Teil der Sammlung bilden. Der Verbund aus den insgesamt 430 embryonalen und fetalen Schnittserien und den modellförmigen Repräsentationen eines kleinen Teils derselben ist das wissenschaftliche Lebenswerk des Anatomen und Embryologen Erich Blechschmidt (1904–1992), der von 1942 bis 1973 das Anatomische Institut der Universität Göttingen leitete (Hinrichsen 1992; Männer 2018).

**Figure 1:**
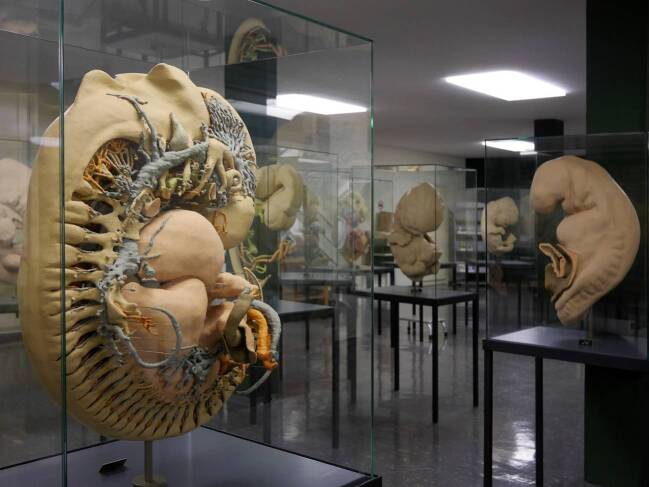

Die Anlage solcher Sammlungen embryonaler Präparate und die Erstellung von körperlichen Modellen auf deren Basis sind die zentralen Topoi der Humanembryologie, wie Nick Hopwood insbesondere für die Sammel- und Modellierpraxis des Anatomen Wilhelm His sowie den Lehrmodellhersteller Friedrich Ziegler mit seinen *Embryos in Wax* – so der Titel einer Monographie Hopwoods (2002b) – herausgearbeitet hat. Während am lebenden Hühnerembryo die Entwicklung schon frühester Entwicklungsstadien, wie etwa das Schlagen des schlauchförmigen Herzens beobachtet werden kann – Aristoteles’ sprichwörtlicher springender Punkt – ist die Embryonalentwicklung des Menschen Beobachtungen weitestgehend unzugänglich. Von der ersten zusammenhängenden visuellen Darstellung menschlicher embryonaler und fetaler Entwicklungszustände durch Samuel Thomas von Soemmering (1755–1830) im ausgehenden 18. Jahrhunderts über Wilhelm His’ (1831–1904) „Normentafel“ der Humanentwicklung von 1885 bis weit in das 20. Jahrhundert war man deshalb auf Sammlungen von Embryonen angewiesen. So trug His über etwa zwei Dekaden insgesamt etwa 80 Präparate meist aus Spontanaborten zusammen, mit denen er jene embryonale Entwicklungskontinuität für den Menschen rekonstruierte, die bei Vögeln, Fischen oder Amphibien leicht zu beobachten war und ist.

Morphologische Details der komplexen embryonalen Entwicklungsvorgänge lassen sich jedoch weder am lebenden Embryo, noch an einer histologischen Schnittserie desselben in hinreichender Weise darstellen. Für ein Verständnis der Lagebeziehungen zwischen Organen und deren dreidimensionale Ausformung wurden deshalb ab Mitte des 19. Jahrhunderts aus den Schnittserien körperliche Repräsentationen abgeleitet, an denen sich Entwicklungsprozesse räumlich nachvollziehen und charakterisieren ließen. Um die Wende zum 20. Jahrhundert erreichten solche Modelle als Lehrmittel aus dem Freiburger Atelier von Friedrich Ziegler (1860–1936) in der Humanembryologie einen hohen Detaillierungsgrad, wie insbesondere die „Serie 3a“, das knapp 40 cm lange und 44 cm hohe, mehrteilige Modell eines menschlichen Embryos am Ende der vierten Entwicklungswoche zeigt (Markert 2019b: 78–79). Forschungsmodelle wurden dazu als serielle Produkte für die medizinische Hochschullehre mobilisiert, denn die Embryologie war zu diesem Zeitpunkt fest im medizinischen Curriculum verankert. Diese Relevanz hatte zwei zentrale Ursachen: Einerseits gerieten durch die zunehmende Medikalisierung von Schwangerschaft embryologische Wissensbestände insbesondere zur Abgrenzung von Fehlbildungen gegenüber einer ‚normalen‘ Entwicklung in den Blick. Vor allem aber profitierte die deskriptive Embryologie vom Diskurs um die Evolutionstheorie und von der Frage, wie viel Wirbeltierevolution in der Embryonalentwicklung des Menschen steckt (Hopwood 2009).

Während die vergleichende Entwicklungsbiologie und die seinerzeit auf den Menschen (noch) nicht anwendbare experimentelle Embryologie von den 1920er bis zu den 1940er Jahren weltweit einen beständigen Aufschwung erlebten, entwickelte sich die auf umfangreiche Sammlungen menschlicher Embryonen aufbauende und in der Medizin verankerte Humanembryologie zu einer Randerscheinung mit mehr curricularer als epistemischer Bedeutung. Spätestens mit neuen bildgebenden Verfahren wie dem Ultraschall, aber auch experimentellen Techniken wie der In-vitro-Fertilisation, wurden innerhalb der Entwicklungsbiologie individuelle, embryonale Schnittserienpräparate mit ihren körperlichen Modellen als Forschungsgegenstand von anderen Entitäten verdrängt (O’Rahilly 1988). Aus dieser Perspektive handelte Blechschmidt mit seinem bis Ende der 1960er Jahre währenden, überregionalen Sammelprogramm menschlicher Embryonen und der mit enormen Ressourceneinsatz betriebenen Erstellung eines in Dimension und Detailreichtum weltweit einmaligen Bestandes an Modellen für Forschung und Lehre gewissermaßen gegen den disziplinären Trend.

Um den Merkmalen von Blechschmidts anachronistischem Forschungs- und Lehrprogramm auf die Spur zu kommen, wird die Humanembryologie als „collecting science“ verstanden, als Disziplin also, in der Sammeln eine „essential practice“ (Kohler 2007: 430) darstellt und die deshalb vorrangig anhand der eingesetzten Praktiken zu beschreiben ist. Dafür werden in der folgenden Analyse die beiden zentralen Praktiken der Humanembryologie fokussiert: das Sammeln menschlicher Embryonen und das darauf aufbauende Modellieren für Forschung und Lehre. Es ist danach zu fragen, was bei der „Dislozierung“ der Sammlungsobjekte und der damit verbundenen „Substitution von Ordnungszusammenhängen“ (Strohschneider 2012: 17) in der Göttinger Anatomie geschah. Wie erfolgte die Sammlungsintegration? Welche Ordnungsprinzipien lagen der Sammlung zugrunde? Welche Bedeutung erhielten die Embryonen durch Ihre Integration in die Sammlung? Mit den Modellierungspraktiken wiederum lässt sich die instrumentelle Dimension der Sammlung beleuchten. Wie Mahr ausführt, haben Modelle eine Doppelfunktion und sind sowohl „Modell von etwas“ als auch „Modell für etwas“ und damit gleichermaßen deskriptiv wie präskriptiv, was sie „in den Wissenschaften zu einer der wichtigsten Arbeitsgrundlagen“ macht (Mahr 2003: 78). In historischer Perspektive wird mit der deskriptiven Dimension auf den Entstehungs-, mit der präskriptiven auf den Verwendungszusammenhang verwiesen. Wovon und in welcher Weise sind die Göttinger Modelle Repräsentationen? Welche Ziele wurden mit der Modellierung verfolgt und welche Verwendungsweisen sind damit verbunden?

Als Beitrag zu einer Analyse der materiellen Kultur der Humanembryologie wird dabei eine heute in Forschung und Lehre weitestgehend durch digitale Verfahren (Chekrouni et al. 2020; Miyazaki et al. 2018; Yamaguchi & Yamada 2018) abgelöste Technologie charakterisiert. Die Mehrdeutigkeit der Dinge korrespondiert dabei „auch in pädagogischer Hinsicht mit der Vielschichtigkeit der Kontexte, die sich situativ verändern und überlagern können“ (König 2012: 25). Wie gezeigt werden wird, führten diese Kontexte in der Göttinger Sammlung weit über das recht enge Feld der Humanembryologie hinaus. Die Modelle entwickelten nach Blechschmidts Ausscheiden gewissermaßen ein Eigenleben, aber ganz in dessen Sinne. Sie wurden dabei vor allem als „strategic objects of knowledge“ (Hopwood & de Chadarevian 2004: 12) sichtbar, denn zusätzlich zur Nutzung in der anatomischen Lehre wird die Modellaufstellung bis in die Gegenwart als materieller ‚Beweis‘ dafür rezipiert, dass schon jüngste embryonale Entwicklungsstadien ganz Mensch und vor Schwangerschaftsabbrüchen zu schützen sind.

Durch die Einbindung der Göttinger Modelle in die breite gesellschaftliche Debatte um den Schwangerschaftsabbruch während der 1980er und 1990er Jahre (Ritter 1991b, 1991a), gerieten auch die gesammelten embryonalen Präparate als Referenzobjekte in den Blick. Blechschmidt selbst machte in seinen Publikationen kaum Aussagen über die Gewinnung und konkrete Herkunft derselben. Dies führte vor allem in Anbetracht eines Forschungsbeginns während des Nationalsozialismus und der Verwendung von 130 Leichen von NS-Opfern vorrangig in anatomischen Kursen von 1942–1944 (Ude-Koeller et al. 2012) zu Spekulationen über ethische Grenzüberschreitungen auch bei der Sammlung von Embryonen und Feten (Ritter 1991b, 1989; Dahms et al. 1998; Mildenberger 2016b, 2016a). Im Rahmen des dazu vom Verfasser 2017–2019 durchgeführten Provenienzforschungsprojektes konnten diese Vermutungen weitestgehend entkräftet werden (Markert 2019a, o.J.). Durch dieses Projekt wurden Archivalien und historische Objekte im institutionellen Umfeld der aus Schnittserien- und Modellteil bestehenden Blechschmidt-Sammlung (o. A. 2020) verfügbar oder mit neuer Perspektive gesichtet, die eine detaillierte Rekonstruktion der Göttinger Sammlungs- und Modellierungspraktiken erlauben. Obgleich bislang kein offizieller Sammlungsbestandteil, wurden diese Materialien der Sammlungsdokumentation hinzugefügt und finden sich detailliert in der folgenden Endnote aufgeschlüsselt.^1^

Die Analyse der Sammlung Blechschmidt erfolgt in fünf Schritten. Zuerst wird die Humanembryologie mit ihren in der zweiten Hälfte des 19. Jahrhunderts etablierten Praktiken eingeführt. Anschließend wird das besondere Lehrkonzept vorgestellt, dass Blechschmidts Aktivitäten zugrunde lag, um davon ausgehend die eingesetzten Praktiken des Sammelns und Präparierens sowie Modellierens zu charakterisieren. Der Einsatz der Modellsammlung in Lehre und Vermittlung dient dann als Ausgangspunkt für eine abschließende Bestimmung von Schnittserien- und Modellsammlung als ein wirkmächtiges Gefüge, das mit disziplinären Praktiken zwar verschränkt, auf diese jedoch nicht reduzierbar ist.

## Humanembryologie als sammelnde und modellierende Wissenschaft im frühen 20. Jahrhundert

Das Forschungsfeld einer dedizierten Humanembryologie ist im Grenzbereich von medizinischer Anatomie und Entwicklungsbiologie zu verorten. Wie Nick Hopwood differenziert und umfassend dargelegt hat, erfolgte ihre Etablierung als eigenständige Disziplin ausgehend vom ursprünglich Basler und später Leipziger Anatomen Wilhelm His in der zweiten Hälfte des 19. Jahrhunderts (vgl. Hopwood 1999, 2002a, 2004, 2007). Bekannt ist His heute vor allem als streitbarer Kollege Ernst Haeckels (1834–1919) in der Auseinandersetzung um dessen vergleichende Darstellung von Wirbeltierembryonen zuerst in der *Natürlichen Schöpfungsgeschichte*. Während Haeckel in seinen Abbildungen die morphologische Ähnlichkeit im Sinne seines evolutionären Standpunkts als eine Rekapitulation der Phylogenese in der Ontogenese betont, interessiert sich His für die Differenz. Hopwood argumentiert, dass His die Embryonalentwicklung der Art Mensch als zu spezifisch und komplex ansah, um sie in ein allgemeines Argument der Wirbeltierentwicklung im Sinne Haeckels zu integrieren (Hopwood 2015: 119–126).

Seine Forschungsarbeit sollte His für die sich formierende Humanembryologie einschlägig machen (vgl. Hopwood 2000; O’Rahilly 1988): Er baute erstens eine Sammlung von Präparaten menschlicher Embryonen mit dem Ziel auf, die Entwicklung des Menschen während der ersten acht Wochen so lückenlos wie möglich zu dokumentieren. Dafür trug His mit Unterstützung eines großen Netzwerks von Ärzten und Hebammen über mehrere Jahrzehnte hinweg eine Sammlung von 79 histologischen Schnittserien menschlicher Embryonen zusammen (Hopwood 2002a). Er führte zweitens mit dem Mikrotom eine Schlüsseltechnologie ein, die das Schneiden von in Paraffin eingebetteten Präparaten als Voraussetzung für kontinuierliche histologische Schnittserien erlaubte. Drittens entwickelte His technisch anspruchsvolle graphische Darstellungsmethoden (His 1887: 385–386; Peter 1906: 77–89) und leitete daraus körperliche Modelle ab, da sich Organstrukturen und ihre Lagebeziehungen mit bloßer Durchsicht der meist zehn Mikrometer starken Schnitte nicht mehr nachvollziehen ließen. Diese Modelle waren nicht nur Forschungs-, sondern auch Lehrgegenstand: Seine im Freiburger Atelier für wissenschaftliche Plastik von Ziegler in Serie produzierten Wachsmodelle (Hopwood 2002b) zur embryonalen Entwicklung des Huhns und des Menschen wurden weltweit in der Hochschullehre eingesetzt und werden bis heute vertrieben (SOMSO MODELLE GmbH 2020). Schließlich formulierte His auf Grundlage seiner Sammlung und der Modelle als Repräsentationen individueller Präparate sowie zugleich Zeitschnitten der embryonalen Entwicklung des Menschen eine erste umfassende Charakterisierung derselben in drei Bänden (His 1880, 1882, 1885). Mit darin enthaltenen „Normentafel“ unterteilte er die Entwicklung vom Ende der zweiten Entwicklungswoche bis zum Ende des zweiten Monats in 25 anatomisch-morphologisch begründete Abschnitte. Damit wurde eine Zuordnung einzelner Präparate zu distinkten Zeithorizonten und damit eine Einordnung in ein als universell verstandenes Entwicklungskontinuum möglich (Hopwood 2000: 31).

His’ Vorgehen – erstens die gezielte Anlage von Sammlungen menschlicher Embryonen, die zweitens als kontinuierliche Schnittserien histologisch aufbereitet wurden und drittens als Modellvorlagen dienten, um daran viertens die ‚normale‘ embryonale Entwicklung des Menschen darstellen zu können – sollte das Fachgebiet bis zur Mitte des 20. Jahrhunderts prägen (O’Rahilly 1988). Geographisch verlagerte sich der Forschungsschwerpunkt dabei in die USA, wo vom His-Schüler Franklin P. Mall (1862–1917) Anfang des 20. Jahrhunderts am *Department of Embryology* der *Carnegie Institution of Washington* die bis heute weltweit bedeutendste Referenzsammlung für frühe humanembryologische Präparate (im Folgenden kurz *Carnegie Collection*) etabliert wurde.

Die Entstehung dieser Sammlung, ihre Rolle für die Embryologie und nicht zuletzt auch für den gesellschaftspolitischen Diskurs über ungeborenes Leben in den USA des 20. Jahrhunderts wurde von Lynn M. Morgan in *Icons of Life. A Cultural History of Human Embryos* (2009) ausführlich dargestellt. Morgan charakterisiert darin eine geradezu industrielle Präparateproduktion, in der Mall und dessen Nachfolger George Linius Streeter (1873–1948) ein sich über die gesamten USA erstreckendes Netzwerk aus Kliniken und niedergelassenen Fachärzt*innen entwickelten und pflegten. Der Aufbau der Kontakte erfolgte durch regelrechte Marketingkampagnen über personalisierte Briefe oder auch Anzeigen in Fachzeitschriften. Stimmten Ärzt*innen einer Mitarbeit zu, so erhielten sie vorbereitete Versandgefäße nebst Einbettungsanleitung, für die das *Department of Embryology *das Porto übernahm. Durch enge Kontakte zum Johns Hopkins Hospital und anderen Kliniken Baltimores konnten Mall und seine Mitarbeiter*innen gelegentlich lebendige Embryonen direkt an der Tür des Operationssaals in Empfang nehmen und selbst fixieren, was eine hohe Präparategüte sicherstellte (ebd.: 76). Allein im Jahre 1919, auf dem Scheitelpunkt der Sammlungsentwicklung, wurden 760 Embryonen und Feten in Malls Sammlung inventarisiert (ebd.: 193), Ende der 1930er Jahre und damit auch gegen Ende der humanembryologischen Sammelaktivitäten dieser Institution lagern dort annähernd 10.000 Präparate.

Mit der Intensivierung und Ökonomisierung der Präparateproduktion um 1900 ging eine Veränderung des Modellbaus einher. His formte seine Modelle seinerzeit freihändig aus Wachs oder Ton und kontrollierte die Maßhaltigkeit in Relation zu den Schnitten mit einen Tasterzirkel, ein Verfahren, welches seinen Zeitgenossen zufolge extrem umfangreiche Vorarbeiten erforderte, zahlreiche Fehlerquellen barg und entsprechend ressourcenintensiv war (Peter 1906: 95). Ab Ende der 1880er Jahre wurde eine neue und weniger anspruchsvolle Technik verfügbar: die sogenannte Wachsplattenrekonstruktionsmethode des Breslauer Anatomen Gustav Born (1851–1900). Dieser beschrieb 1883 und 1888 seine „Plattenmodellirmethode“ (Born 1883, 1888), bei der histologische Schnittserien von Wirbeltierembryonen maßstabsgerecht vergrößert erst auf Zeichnungen und dann auf eigens dafür gegossene Wachsplatten von meist ein bis zwei Millimeter Stärke übertragen wurden.^2^ Stapelte man nun die ausgeschnittenen Formen entlang einer zuvor markierten Richtebene aufeinander, so erhielt man ein wächsernes Modell ebenjener Anatomie, die sich zuvor über Duzende oder gar Hunderte mikroskopische Einzelschnitte und damit Einzelbilder erstreckte. Vermessung und anatomische Beschreibung erfolgten dann am Modell, das als eine morphologisch identische, wenn auch vergrößerte Repräsentation eines individuellen embryonalen Präparats verstanden wurde. Wie der experimentelle Embryologe Wilhelm Roux (1850–1924) in einem Nachruf auf Born im Jahre 1900 ausführte, war dessen Methode aus der zeitgenössischen Embryologie nach zwei Dekaden nicht mehr wegzudenken:

Diese ist eine universelle, mechanische Rekonstruktionsmethode zur fehlerfreien Übertragung des mikroskopisch Kleinen ins Makroskopische, die die natürliche Ergänzung des Mikrotomirens darstellt, indem das durch letztere Methode lückenlos Aufgeschlossene durch erstere lücken- und fehlerlos, mechanisch […] ins Große übertragen wird. Durch beide zusammen wurde es möglich, dass jetzt sogar jeder Ungeübte und Unbeanlagte schwierige Details der Entwickelungsvorgänge genauer wahrnehmen kann, als es früher die allerbesten Beobachter, wie Baer und Pander mit den Mitteln ihrer Zeit zu thun im Stande waren. (Roux 1900: 259)

Sehr deutlich klingt hier auch an, was schon Born beschrieb, nämlich dass man für bestimmte Arbeitsschritte des Verfahrens „jeden intelligenten Diener dazu rasch abrichten“ könne (Born 1883: 599). Das zweifellos arbeitsintensive, aber eben auch schlichte und damit arbeitsteilig ausführbare mechanische Verfahren war innerhalb kürzester Zeit in zahllosen Versionen im Einsatz (Peter 1906; Strasser 1887) und dies zudem weltweit: Als die Embryologin Susanna Phelps Gage (1857–1915) im Jahre 1907 vorschlug, aus Zeit- und Kostengründen die Wachsplatten durch Pappe zu ersetzen (ähnlich schon His 1887: 388–389), konnte sie für den amerikanischen Raum festhalten, was in Europa schon längst galt: „The Born Method of reconstructing models from wax plates is in use in all the larger laboratories of Anatomy and Embryology.“ (Gage 1907: 166)

An der *Carnegie Collection* entstanden allein durch den Modellbauer Osborne Overton Heard von 1913 bis 1956 über 800 Wachsplattenmodelle für Forschungszwecke (Wellner 2014). Sie dienten wie seinerzeit die His’schen Modelle vor allem zur Charakterisierung normaler Embryonalentwicklung, die in den 1942 und 1948 von Malls Nachfolger George Linius Streeter herausgeben „developmental horizons“ mit 23 anatomisch spezifischen Entwicklungszuständen des menschlichen Embryos mündeten (Hopwood 2007: 22–23). Mit dem Abschluss dieser bis heute angewandten, wenn auch in den Details mehrfach überarbeiteten (O’Rahilly 1988: 101–102) Charakterisierung embryonaler Entwicklungsphasen verlagerte sich das Forschungsprogramm des *Department of Embryology* weg von der Sammlung und den daran darstellbaren Fragen (Hopwood 2007: 23) hin zu künstlicher Befruchtung, Hormonforschung Plazentologie, Teratologie und vergleichender Embryologie. Deutlich benennt 1957 der damalige Leiter des *Departments* im Jahresbericht der *Carnegie Institution* James David Ebert (1921–2001) den inzwischen etablierten Status quo: „It can no longer be stated that our investigations are concerned chiefly with the form and function of the human embryo. [..] this phase of our activities has reached the point of diminishing return.“ (Ebert 1957: 299) Zwar solle die Humanembryologie und damit auch die Sammlung in einer „ancillary position“ erhalten bleiben und weiterhin mit möglicherweise forschungsrelevanten Embryonen vor allem sehr junger Entwicklungsstadien erweitert werden, „[b]ut the challenging problems in embryology today confront the experimentalist.“ (Ebd. 1957: 300)

## Das humanembryologische Programm Blechschmidts

Als Blechschmidt 1942 das Göttinger Anatomische Institut übernahm und mit dem Aufbau einer humanembryologischen Sammlung begann, existierte der von Ebert benannte Trend schon einige Zeit. Aufgrund des beständigen, globalen Aufschwungs der benachbarten Forschungsfelder der experimentellen Embryologie und der vergleichenden Entwicklungsbiologie ab den 1920er Jahren war die sammelnde und modellierende Humanembryologie inzwischen nur noch eine Randerscheinung (Hopwood 2019). An anderen anatomischen Instituten des deutschsprachigen Raums wie in Heidelberg (Doll 2019) oder Wien (Nemec 2019: 89–91) waren in der ‚Hochphase‘ der Humanembryologie um 1900 entsprechende Sammlungs- und Modellierungsprojekte etabliert worden, inzwischen hatte das Thema aber schon wieder deutlich an Bedeutung verloren. Blechschmidt schuf in Göttingen jedoch nicht nur ein neues und anachronistisches Arbeitsgebiet, er konnte dafür in den letzten Kriegs- und ersten Nachkriegsjahren erstaunlich umfangreiche Ressourcen akquirieren. Wie gezeigt werden wird, war dafür der Embryologiemodellbau in einer sehr eigenwilligen Ausprägung von besonderer Relevanz.

Für Erich Blechschmidt wurde an anderer Stelle von Florian Mildenberger eine umfangreiche Biographie mit einem Schwerpunkt auf der NS-Zeit vorgelegt, auf die an dieser Stelle verwiesen sei (Mildenberger 2016a; auch Ude-Koeller et al. 2012), sodass die biographischen Informationen hier auf ein Minimum beschränkt werden können. Blechschmidt wurde am 13. November 1904 in Karlsruhe als Sohn eines Arztes geboren und studierte von 1922 bis 1927 Humanmedizin in Freiburg, München und Wien. Nach seiner Approbation im März 1929 wurde er Assistent am Institut für Anatomie der Universität Freiburg unter dem embryologisch interessierten Ordinarius Wilhelm von Möllendorff (1887–1944), dort 1930 promoviert und 1934 zum 2. Prosektor ernannt. Blechschmidt habilitierte sich 1935 mit einer Arbeit zum *Konstruktionsplan der Neugeborenenlunge *(Blechschmidt 1935) und damit zu anatomischen Aspekten der Humanentwicklung, wie sie in den kommenden Jahren immer stärker seine Forschung prägen sollte (etwa Blechschmidt 1937, 1938, 1939). Im Jahre 1940 vertrat Blechschmidt zuerst die Prosektur in Gießen, danach in Würzburg und ab dem 1. Dezember 1941 übernahm er vertretungsweise die Direktion des Anatomischen Institutes in Göttingen. Am 1. Juli 1942 erfolgte dort die Ernennung zum Direktor und außerordentlichen Professor^3^ und am 13. Juni 1949 zum ordentlichen Professor,^4^ wovon er nach fast 24 Dienstjahren zum 31. März 1973 entpflichtet wurde.^5^

Den bisherigen Recherchen zufolge wurde Blechschmidts Werk innerhalb der Embryologie kaum rezipiert und obgleich Mildenberger (Mildenberger 2016a) eine erste Einordnung vornahm, stehen dort mögliche NS-Verstrickungen Blechschmidts bei der Präparategewinnung im Vordergrund, weniger das entworfene Denkgebäude und seine Anschlussfähigkeit. Wie sein Vorbild His, dem Blechschmidt 1948 seine erste Monographie widmete, stand er der vergleichenden Entwicklungsbiologie in der Tradition Haeckels ablehnend gegenüber und isolierte sich damit gegenüber benachbarten Forschungsfeldern. Die von ihm beschriebene Embryonalentwicklung wird von den mechanischen Kräften Zug und Druck bestimmt, physiologische, genetische und phylogenetische Aspekte werden nicht berücksichtigt. Obgleich Blechschmidt seine erste Monographie *Mechanische Genwirkungen* nannte, spielen Gene und Genetik darin nicht nur keine Rolle: Sie werden gemeinsam mit der Physiologie in der Einleitung als selbstverständlich vorausgesetzt und damit als weitestgehend irrelevant für Blechschmidts mit dem Buch avisierte Bestimmung der „Lebensäußerungen nach formalen sowie nach physikalischen und damit u. a. nach mechanischen Merkmalen“ (Blechschmidt 1948: 1) erklärt.

Der 1933 in Göttingen promovierte und vor Kriegsbeginn in die USA emigrierte Genetiker Ernst Caspari (1909–1988) hält in einer Besprechung des Werkes fest, dass Blechschmidt darin nicht nur in inakzeptablem Maße embryonale Vorgänge auf sein mechanistisches Erklärungsmodell hin vereinfacht, sondern das Buch auch sprachlich eine Herausforderung darstellt: „Particularly disturbing is the author’s tendency to use well-established technical terms, such as induction, determination, synapse, in a different meaning from those customary in biology.“ (Caspari 1950) Blechschmidts Begriffsbildung und konzeptionelle Ausrichtung waren auch für seine Zeitgenossen in der Anatomie so irritierend, dass der Wiener Ordinarius Ferdinand Hochstetter (1861–1954) 1943 an seinen Prager Kollegen Otto Grosser (1873–1951) schrieb, nach der Lektüre von Blechschmidts letzter Abhandlung (Blechschmidt 1941) halte er diesen „geistig für nicht ganz normal.“^6^ Der Würzburger Anatom und langjährige Herausgeber der *Zeitschrift für Anatomie und Entwicklungsgeschichte* Curt Elze (1885–1972) war gefragt nach Kandidaten für die 1946 vakante Münchner Professur zwar wohlwollend, aber ähnlich direkt: „Blechschmidt-Göttingen (1904), ein unübertrefflicher Organisator und Institutsleiter, leidenschaftlicher Arbeiter, voller Ideen, aber von einer nicht zu bändigenden Neigung zum Theoretisieren bis zur Verstiegenheit und Unverständlichkeit.“^7^

Es ist nicht bekannt, ob sich die insgesamt negative fachliche Einschätzung auf die Frühphase von Blechschmidts Schaffen bis zur Übernahme der Professur beschränkt, da entsprechende disziplinenhistorische Untersuchungen bisher ausstehen. Aus der wenig ausgeprägten Rezeption der Arbeit Blechschmidts und einem weitgehenden Fehlen fachwissenschaftlichen Schriftwechsels im Institutsarchiv entsteht allerdings der starke Eindruck, Blechschmidt hätte mit seinem konzeptionellen Zugang zur Embryogenese wenig Anschluss gefunden. Auch Blechschmidts Schüler und langjährigem Mitarbeiter Klaus Volquart Hinrichsen (1927–1997), 1970–1997 Lehrstuhlinhaber für Anatomie und Embryologie in Bochum, fiel es augenscheinlich schwer, das Lebenswerk Blechschmidts inhaltlich zu fassen. In seinem Nachruf auf Blechschmidt in den *Annals of Anatomy* zitierte er diesen zwar mit der angeblichen „Grundthese“: „Der Embryo ist das *natürliche* Schema des Erwachsenen.“ (Hinrichsen 1992: 480) Diese wurde jedoch nicht näher ausgeführt, wie auch andere inhaltliche Aussagen sehr allgemein blieben.

Ausführliche Würdigung fand hingegen an verschiedenen Stellen des Nachrufs die von Blechschmidt forcierte Anschaulichkeit und zwar sowohl in dessen bildgewaltigem wissenschaftlichen Hauptwerk – „die großen Publikationen, die die Rekonstruktionen dokumentieren oder auf ihnen fußen“ (ebd. 1992: 481) – als auch in seinen „eindrucksvollen plastischen Zeichnungen“ (ebd.: 480) an der Hörsaaltafel. Schon seine ansonsten weitgehend vernichtende Rezension der *Mechanischen Genwirkungen* beendete Caspari mit überraschender Begeisterung über die Bildsprache des Buches: „This review would be incomplete without calling attention to the large number of excellent illustrations,“ wobei ihm zufolge weniger die hervorragenden Mikrofotografien als vielmehr die schematischen Zeichnungen für Studierende der Anatomie und Embryologie sehr wertvoll gewesen sein dürften (Caspari 1950).

Tatsächlich prägte das Bemühen um Anschaulichkeit in der medizinischen Ausbildung das Schaffen Blechschmidts von der Berufung bis zur Emeritierung. Kurz vor seiner offiziellen Ernennung zum Direktor der Göttinger Anatomie beantragte Blechschmidt im Juni 1942 Sachbeihilfe bei der Forschungsgemeinschaft der Deutschen Wissenschaft insbesondere für die Arbeit an einem später nicht fertiggestellten Handbuchbeitrag zur „Entwicklung der menschlichen Körperform“, wobei in der Liste der benötigten Geräte auch ein „Apparat zum Walzen von Wachsplatten“ für den Modellbau vermerkt war.^8^ Gegen Kriegsende argumentierte er die weitere Förderung seiner Arbeit mit einer neuen anatomischen Didaktik und erläuterte diese im Juni 1944 dem Präsidenten des Reichsforschungsrates in dem ihm eigenen Duktus:

Die gefundene regelmäßige Lageverschiedenheit zwischen dem lockeren und straffen Bindegewebe zeigt durch ihre zunehmende Deutlichkeit im Laufe der Entwicklung an, dass bestimmte Massenbewegungen, also mechanische Vorgänge des wachsenden Körpers die unmittelbare Ursache für die Gestaltung der äusseren Körperform sind. Hiermit ist die Grundlage für eine bisher noch nie systematisch bearbeitete Kausale Morphologie festgestellt. […]

Da sich im Laufe meiner fünfstündigen Vorlesung des gegenwärtigen Sommersemesters gezeigt hat, dass die Darstellung der Kausalen Morphologie des Bewegungsapparates eine unerwartete Vereinfachung in der Beschreibung der Form und Anordnung und im besonderen der Ansatzverhältnisse der Muskeln ergibt, erscheint mir die vorzugsweise Bearbeitung dieses Teilgebietes geboten.^9^

Was Blechschmidt hier erstmals formulierte und in den folgenden Jahren vorantrieb, ist ein innovatives anatomisches Lehrkonzept, in dem die Morphologie des Erwachsenen konsequent auf die embryonale Morphologie zurückgeführt wird – allerdings geschah dies in einer fachwissenschaftlich durchaus fragwürdigen Reduktion aller Entwicklungsprozesse auf einfache „Massenbewegungen“. Im Jahre 1951 berichtete Blechschmidt einem spanischen Kollegen, der für den Neuaufbau eines anatomischen Instituts europaweit Ideen für Lehrkonzepte einholte:

In den Vorlesungen, in denen große Modelle menschlicher Embryonen stehen, wird betont, daß zwar der menschliche Leichnam in Bauelemente zerlegt werden kann, daß aber der wirkliche Organismus nicht durch Zusammensetzen von Bauklötzen entstanden ist, sondern tatsächlich ein ‚Entwicklungsprodukt der Eizelle‘ darstellt. […] Ich weiß, daß hier für uns Dozenten erst der Anfang gemacht ist und eine riesige Arbeit bevorsteht; aber das Anfangen macht auch unseren Studenten eine größere Freude, als das langweilige Auswendiglernen längst veralteter Dinge.^10^

Ein wesentlicher Teil des angesprochenen Arbeitsaufwands dürfte für Blechschmidt das sich anschließende Projekt einer umfassenden Repräsentation der menschlichen Embryonalentwicklung in dutzenden, extrem detaillierten und etwa 75 cm hohen Kunststoffmodellen gewesen sein, dass gegen Mitte der 1960er Jahre abgeschlossen wurde.

## Die Entwicklung der Präparatesammlung als Voraussetzung der Modellbildung

Mit einer Fokussierung auf die vergrößerten (Lehr‑)Modelle als Repräsentationen abstrakter embryonaler Entwicklungsstufen und deren anatomisch-morphologischen Details gerät leicht aus dem Blick, dass dafür histologisch aufbereitete menschliche Embryonen als Quelle für die nötigen Umzeichnungen (s. S. 19) und dauerhafte Referenz in einer Sammlung vorliegen müssen. Tatsächlich begann Blechschmidt direkt nach Antritt der Institutsleitung in Göttingen im Jahre 1942 nicht nur mit dem embryologischen Modellbau – etwa nach bereits publizierten Schnittserien aus fremden Sammlungen – sondern auch mit dem Aufbau eines eigenen Embryonenbestandes. Ein erstes Konvolut von 29 menschlichen Embryonen, vor allem aber entwicklungsälteren Feten wurde ihm im Februar 1943 vom Leiter der damaligen Gaufrauenklinik in Posen, Boris Belonoschkin (1906–1988) zugesandt.^11^ Zu Beginn des Jahres 1944 etablierte Blechschmidt zudem die Göttinger Universitätsfrauenklinik als dauerhafte Quelle für „Embryonen beliebiger Größe“ sowie „ausgetragene Früchte“ für histologische Untersuchungen durch persönliche Vereinbarung mit Klinikleiter Heinrich Martius (1885–1965).^12^ Ebenfalls erfolgreich waren zeitnahe Anfragen an die Landesfrauenkliniken in Hannover und Celle^13^ sowie das Klinikum in Bad Lauterberg am Harz, von wo den Einbettungsprotokollen zufolge mindestens neun der 18 im Jahre 1944 eingebetteten Präparate stammen.

Nach Kriegsende intensivierte Blechschmidt die Aktivitäten, verschickte personalisierte Anschreiben an vorrangig niedersächsische Kliniken^14^ und warb in Fachzeitschriften für sein Sammelprogramm (o. A. 1949, 1957). Unter den einsendenden Ärzt*innen waren ehemalige Studierende Blechschmidts aus dessen Freiburger Zeit, so in Salzgitter und Oldenburg.^15^ Auch als Famuli oder Assistenzärzt*innen tätige Student*innen aus Göttingen gehörten zu den Sender*innen oder vermittelten entsprechende Kontakte.^16^ Sowohl in den personalisierten Anschreiben als auch den gedruckten Anzeigen argumentierte Blechschmidt eine Beteiligung an seinem Sammelprogramm unter anderem damit, dass die in der Klinik ohnehin anfallenden Embryonen sonst für Forschung und Lehre verlorengingen (o. A. 1957).

Blechschmidt ermunterte die Einsendenden dabei immer wieder, ihm auch eher ungeeignete Präparate zu schicken. Er nahm „gern viele Versager in Kauf, wenn nur die Hoffnung besteht, daß irgendwann einmal etwas gutes anfällt.“^17^ Zudem wurde „weniger wertvolles Material“ etwa „beim Ausprobieren neuerer Methoden“^18^ benötigt. Dabei war der „Embryonenhunger“ im Göttinger Institut, so Blechschmidt 1944 wörtlich an Belonoschkin,^19^ kaum stillbar. Noch Ende der 1950er Jahre, als Blechschmidts bis heute im Institut aufgestellten Modelle weitestgehend abgeschlossen und alle dafür relevanten Schnittserien vorhanden waren, schrieb er einem Einsender: „Es besteht hier immer Bedarf an frischen Embryonen.“^20^

Auf diese Weise konnte Blechschmidt im Zeitraum von 1942–1969^21^ mehrere Hundert Personen aktivieren, mit deren Unterstützung er innerhalb von 27 Jahren eine Sammlung mit heute 430 Schnittserien von 116 vollständigen Embryonen mit Körperlängen von 2,57–64 mm und Körperteilen von etwa 170 Feten aufbaute. Die Hochphase der Aktivitäten war dabei der Zeitraum von 1947–1954, in dem laut Inventar 51 Prozent der heute vorhandenen embryonalen und fetalen Schnittserien bzw. 69 Prozent aller Schnittserien der besonders wertvollen ganzen Embryonen in die Sammlung gelangten. Insgesamt dürften mehrere Tausend Embryonen und Feten in Blechschmidts Institut eingegangen sein. Aufgrund unzureichender Fixierung, pathologischer Merkmale oder den Gewinnungsumständen hatten sie jedoch oft nicht die Qualität für eine histologische oder makroskopische Aufbereitung zur dauerhaften Integration in eine Sammlung zur embryonalen Normalentwicklung oder einen Einsatz in der Lehre.

Die konkreten klinischen Vorgänge der Präparategewinnung erlauben einen Einblick in den ‚ersten Ordnungszusammenhang‘ (Strohschneider) der Präparate – den Leib einer Schwangeren – und umfassten ein Spektrum, dass auch für andere Sammlungen wie die *Carnegie Collection* nachweisbar ist (Morgan 2002: 265). Ein Teil der Embryonen stammt als ‚Zufallsfunde‘ sowohl aus Spontanaborten als auch Leichenschauen – so das entwicklungsjüngste Präparat der Sammlung, „Embryo/Ei 2,5 mm, 28.11.1950“.^22^ Zuverlässige Quellen waren zudem verschiedene Operationsformen, etwa Hysterektomien, das heißt die operative Entfernung des Uterus, sowie Extrauteringraviditäten. Gerade Eileiterschwangerschaften traten nach dem zweiten Weltkrieg und bis in die 1950er Jahre – und damit parallel zur Hochphase der Blechschmidt’schen Sammelaktivität – in Deutschland gehäuft auf, was in der entsprechenden epidemiologischen Fachliteratur mit Gonorrhoe als Grunderkrankung und davon ausgelösten Tubenveränderungen in Verbindung gebracht wird (Heberer 1948; Lehmann 1951; Schramm 1952). Zuletzt kamen auch Präparate aus freiwilligen^23^ Schwangerschaftsabbrüchen in der Nachkriegszeit und erzwungenen^24^ während der NS-Zeit in Frage.

Die Patientinnen als eigentliche Präparatequellen spielen mit ihren Fallgeschichten in der überlieferten Korrespondenz des Sammelprogrammes eine sehr marginale Rolle. Soweit bekannt, wurden Patientinnen auch im Rahmen vergleichbarer humanembryologischer Sammelprogramme meist weder um ihr Einverständnis gebeten noch über die Weiterverwendung ‚ihrer‘ Embryonen und Feten informiert. Während jedoch andere Sammler der Zeit (etwa Rosenbauer 1955: 236), aber auch zuvor Mall und seine Kollegen (Morgan 2004), umfangreiche Daten zu Anamnese, Diagnose und Therapie vorhielten, finden sich entsprechende Informationen bei Blechschmidt nur selten in den Protokollen oder Inventarverzeichnissen. Zudem bittet Blechschmidt mit der Begründung der Arbeitsersparnis für die ärztlichen Kooperationspartner*innen gelegentlich darum, auf Begleitbriefe zu verzichten, da diese ihm zufolge für den ‚Sammelwert‘ eines Präparates keine Rolle spielen:

Da es für die praktische Untersuchung nur auf den Erhaltungszustand bzw. die Größe der Embryonen ankommt, d. h. also auf Ergebnisse, die sich immer erst hinterher nach der Aufarbeitung der Embryonen genauer bestimmen lassen, so schadet es nichts, wenn Sie künftighin die Exemplare vereinfachterweise ohne jedes Begleitschreiben absenden.^25^

Der überlieferte Briefwechsel vermittelt als Reaktion auf Blechschmidts Anfragen den Eindruck kollegial-professioneller Kontakte zur Unterstützung eines wissenschaftlichen Forschungsprogramms, wobei die damit verbundene Einsatzbereitschaft eine große Bandbreite aufweist. Manche Ärzt*innen schickten als Reaktion auf einen öffentlichen Aufruf ein Präparat, danach brach der Kontakt wieder ab.^26^ In vielen Fällen jedoch bestanden Verbindungen über einige Monate oder auch Jahre und in Einzelfällen waren geradezu enthusiastische Einsender darunter. Ein Mediziner aus Salzgitter, der in den 1960er Jahren mindestens ein Dutzend Präparate schickte, schrieb in seinem Begleitbrief zu einem Spontanabort: „Am 01.10.1965 werde ich diese Abteilung hier selbst übernehmen und werde dann noch besser in der Lage sein, Sie zu versorgen. Ich komme mir vor wie ein Holzlieferant, der einen Bildhauer beliefert – aber ohne Holz wäre ja auch der Bildhauer schlecht dran!“^27^

## Das Göttinger Modellierungsverfahren

Für die Umwandlung des „Holzes“ eines liefernden Arztes in ein Modell durch den Bildhauer Blechschmidt – oder genauer das Modellbauteam mit Blechschmidt als Mäzen – wurde in Göttingen eine eigens entwickelte Variante des Born’schen Verfahrens eingesetzt. Obgleich Born ursprünglich seine „Plattenmodelirmethode“ für Wachs als Werkstoff beschrieb, wurde schon wenige Jahre nach Einführung des Verfahrens mit Platten etwa aus Papierwerkstoffen oder Glas ebenso experimentiert, wie mit Gips- und Metallausgüssen in Negativformen, um dauerhafte, stabile Modelle herzustellen (etwa Selenka 1886). An der Göttinger Anatomie entstand ein Jahr nach Blechschmidts Übernahme der Institutsleitung eine Doktorarbeit zur *Formentwicklung des menschlichen Nierenbeckens* gegen Ende der Embryonalentwicklung, für die Ausgussmodelle des Ureterbäumchens aus Marmorzement konstruiert wurden (Sonntag 1943: 665). Die Qualität solcher Ausgussmodelle allerdings stellte Blechschmidt, der in den 1940er Jahren auch mit Plexiglasplatten und Gelatine als Modellmaterial experimentierte,^28^ vor allem wegen deren Detailarmut nicht zufrieden (Blechschmidt 1954: 170).

**Figure 2:**
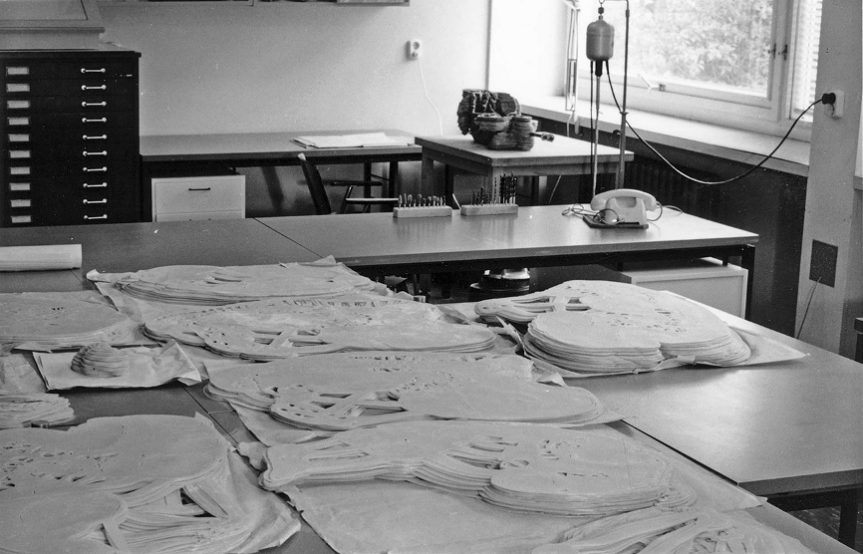

Mit dem Verfügbarwerden von Polyesterharz als festem, leichtem und je nach Zusammensetzung auch bei Zimmertemperatur und damit unterhalb des Schmelzpunktes von Wachs aushärtendem Werkstoff ab Ende der 1940er Jahre fanden Versuche zum Einsatz im Rekonstruktionsmodellbau statt – in Göttingen, aber auch andernorts (etwa Boyer 1948). Wie Blechschmidt in einer Publikation seiner Variante des Verfahrens im Jahre 1954 beschreibt, strich man an der Göttinger Anatomie eine teigige Masse – bestehend aus dem von Bayer produzierten Kunstharz *Leguval* mit dem nötigen Härter, etwas Farbpulver für die Differenzierung von Organsystemen und einer größeren Menge Gips oder Quarzmehl als Füllstoff – in Platten aus Paraffinwachs von einem bis zwei Millimeter Stärke ein (Abb. 2). Nach dem vollständigen Aufbau der Modellkörper wurde das umgebende Wachs unter heißem Wasser abgeschmolzen, das Modell geschliffen, verspachtelt und nachkoloriert, wobei teilweise die Modelle auch wieder aufgeschnitten werden mussten, um das Wachs aus unzugänglichen Hohlräumen auszuschmelzen. Die Herstellungsdauer jedes der heute noch ausgestellten Modelle mit einer Höhe von etwa 75 cm gibt Blechschmidt mit mehreren Wochen an – wohlgemerkt nur für die schichtweise Konstruktion. Ebenfalls mehrere Wochen sollen jeweils die photographischen und zeichnerischen Vorbereitungen sowie die Nacharbeiten zur Oberflächenbehandlung in Anspruch genommen haben (Blechschmidt 1954: 173), wobei einem Zeitzeugen zufolge in diese Arbeiten durchgängig drei bis vier Personen involviert waren.^29^

**Figure 3:**
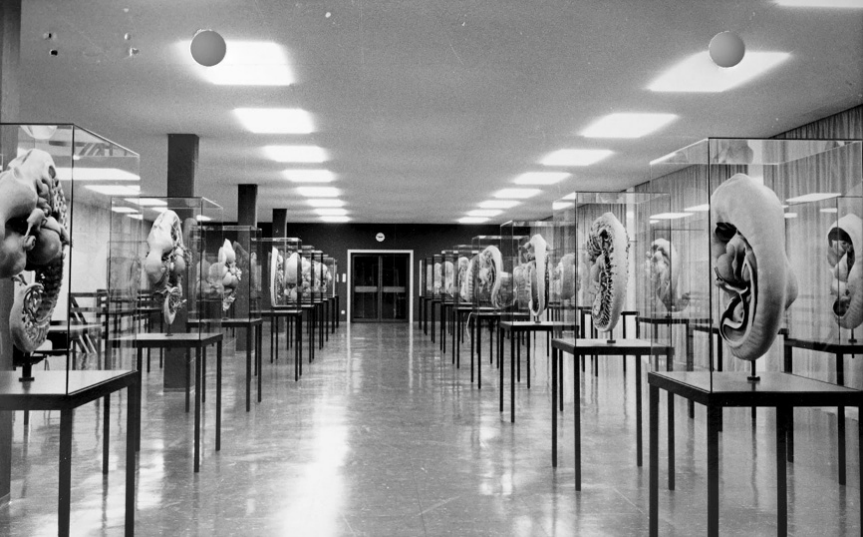

Obgleich sehr frühe Entwicklungsstadien nach Präparaten in anderen Sammlungen modelliert wurden, geht der überwiegende Teil der in Göttingen ausgestellten Modelle auf einige wenige lokale Schnittserien zurück. Insgesamt 40 der heute 61 Modelle, darunter die 31 sogenannten Totalrekonstruktionen ganzer Embryonen von Kopf bis Steiß, wurden nach nur neun Schnittserien erstellt. Die ursprünglichen Körperlängen dieser Präparate, die von 1945 bis 1954 ans Institut gelangten, betrugen 2,57 mm, 3,1 mm, 3,4 mm, 4,2 mm, 6,3 mm, 7,5 mm, 10 mm, 13,5 mm und 17,5 mm, was die Dokumentation der Entwicklung von der dritten bis zur achten Woche nach Befruchtung erlaubt. Zu den einzelnen Schnittserien sind bis zu sieben Einzelmodelle vorhanden, da sich Strukturen im Körper gegenseitig verdecken und daher jedes Organsystem bzw. jeder Darstellungsschwerpunkt ein eigenes Modell erforderte. Überwiegende Teile des Modellbestandes sind wohl zur Eröffnung des Neubaus für die Anatomie am Rande des Göttinger Universitätscampus 1964 abgeschlossen gewesen. Auf einer als Neujahrsgruß zum Jahreswechsel 1964/65 an Präparatesender*innen versandten Fotografie des damaligen Ausstellungsraums (Abb. 3) allerdings sind bestenfalls 40 der heute ausgestellten 61 Modelle zu sehen, sodass einige später entstanden sein dürften.

Die Schlüsselfigur zur Entwicklung des Verfahrens und seiner Umsetzung in zahlreichen Modellen war nicht Blechschmidt selbst, sondern dessen wissenschaftlicher Assistent Willi Kircheiss (Lebensdaten unbekannt). Unter dem 1951 als Grafiker und Modelleur eingestellten Diplom-Ingenieur entstanden mit einer Ausnahme^30^ alle heute in Göttingen präsentierten Rekonstruktionsmodelle menschlicher Embryonen und Feten. Zudem geht Blechschmidt zufolge der Einsatz von Kunststoffen auf Kircheiss zurück,^31^ auch wenn ersterer dies in der Publikation des Verfahrens nicht erwähnt. Genannt wird der Ingenieur hingegen als Modelleur und Zeichner in zwei großen Monographien zur Göttinger Modellsammlung (Blechschmidt 1961: 6, 1963a: Vorwort).

**Figure 4:**
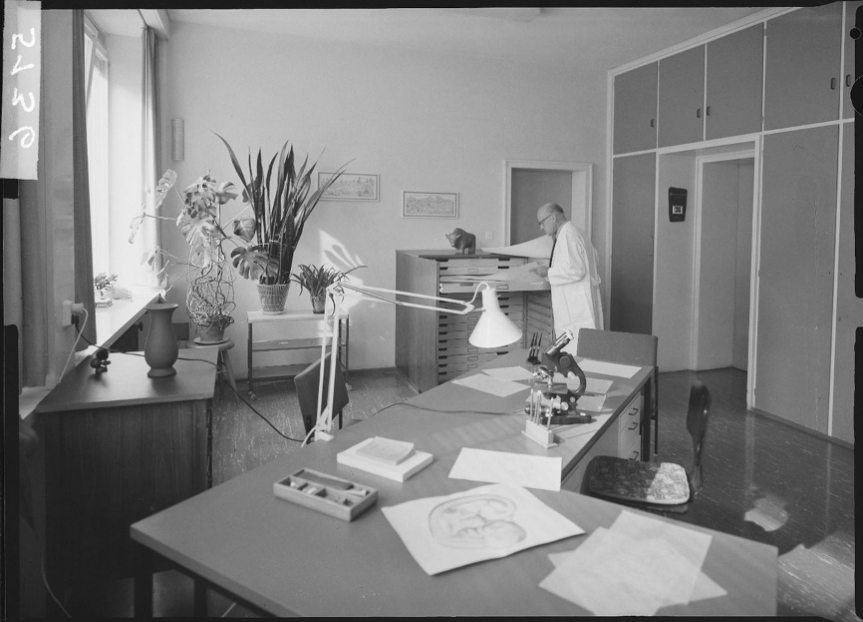

Über Kircheiss ist aufgrund fehlender Personalunterlagen nur wenig bekannt, gleichwohl liegt ein Planfilmnegativ vor, auf dem er von seinem Nachfolger als Institutszeichner identifiziert wird.^32^ Auf dem Bild steht Kircheiss an einem Planschrank, der Zeichnungen für Publikationen Blechschmidts und die Dissertationen von dessen Doktorand*innen enthielt (Abb. 4).

**Figure 5:**
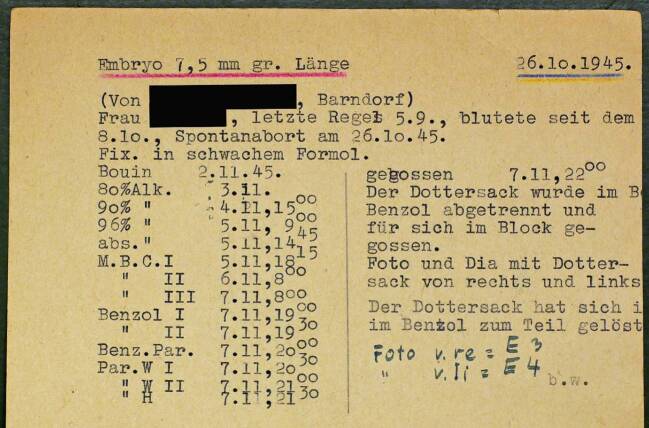

Wie die kunstvollen Großrekonstruktionen für die Dauerausstellung entstanden, lässt sich aufgrund der spezifischen Quellenlage am Modellierungsprozess zum Embryo mit der Bezeichnung „26.10.1945, 7,5 mm“ aus der sechsten Entwicklungswoche besonders gut nachvollziehen: Am 2. November 1945 bestätigte Blechschmidt einem Arzt in einer kleinen niedersächsischen Gemeinde in der Nähe von Vechta den Eingang eines Präparates aus einem Spontanabort.^33^ Wie Blechschmidt dem Einsender mitteilte, habe er „bisher noch nichts in der Größe erhalten können.“^34^ Die Präparation des Embryos durch die technische Assistentin Ilse Waßmann (Lebensdaten unbekannt), die spätestens ab 1944 und bis etwa 1955 für Blechschmidt arbeitete und damit den überwiegenden Teil aller noch vorhandenen Schnittserien anfertigte,^35^ begann zeitnah nach dessen Eingang im Institut am 2. November mit der Umbettung in frische Fixierungslösung (Bouin). Bei dieser Gelegenheit wurde die Körperlänge vermessen und ging üblicherweise gemeinsam mit dem Datum des Präparationsbeginns als Objektbezeichnung in die Sammlungsdokumentation ein. Dass in diesem Fall der Zeitpunkt der Gewinnung des Präparates als Datum festgelegt wurde, dürfte auf einen Übertragungsfehler zurückzuführen sein, denn von nun an hatte das Präparat als Sammlungsobjekt die Bezeichnung „26.10.1945, 7,5 mm“. Während der folgenden vier Tage fand die Vorbereitung des Embryos auf die Umwandlung in eine Schnittserie statt. Erst wurde ihm in einer Alkoholreihe das Wasser entzogen, der Alkohol dann gegen Benzol und dieses endlich gegen Paraffinwachs ausgetauscht (Abb. 5). Nach dem Aushärten konnte das Präparat mit dem Mikrotom in eine lückenlose Serie von über 600 horizontalen bzw. transversalen Schnitten mit einer Stärke von je zehn Mikrometern zerlegt werden.

**Figure 6:**
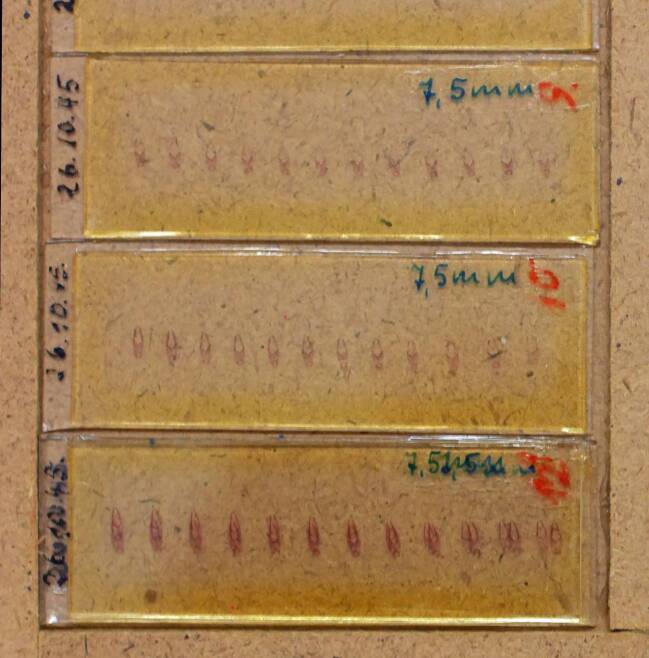

Nach dem Entziehen des Paraffins und einer Azan-Färbung – einer histologischen Standardmethode – erfolgte das Aufbringen der Schnitte auf Objektträger und das Bedeckeln zur dauerhaften Haltbarmachung (Abb. 6).

**Figure 7:**
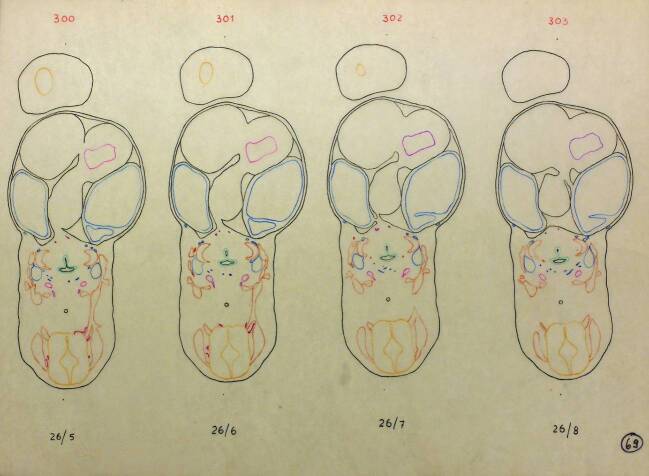

In Vorbereitung des späteren Modellbaus wurde 1949 eine Mappe mit farbig kodierten Zeichnungen aller Einzelschnitte von Embryo 26.10.1945, 7,5 mm angelegt, wobei solche Mappen für alle modellierten Embryonen existieren (Abb. 7). Diese Zeichnungen dienten als Vorlage für vergrößerte Umzeichnungen aus Packpapier, deren Markierungen dann auf Wachsplatten zur Herstellung der Negativform übertragen wurden.

**Figure 8:**
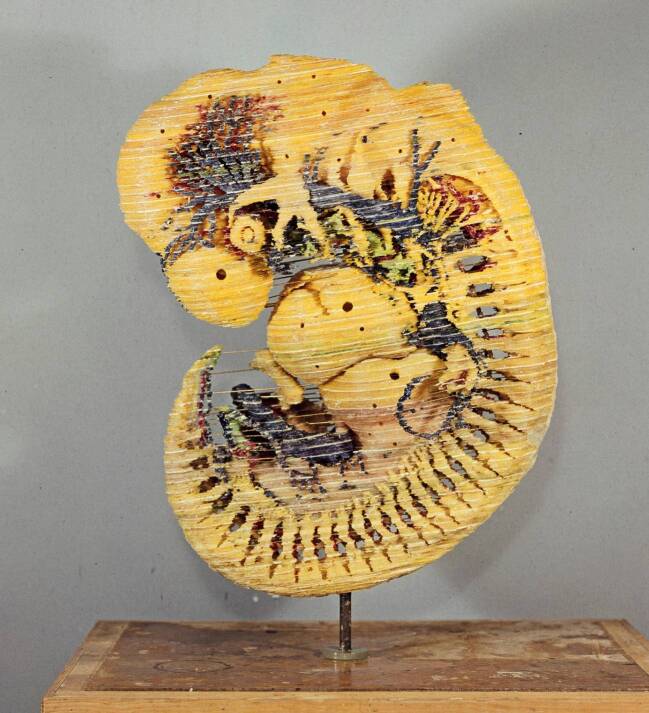

Zum Entstehungsprozess der Modelle des 7,5 mm-Embryo sind farbige Planfilmnegative überliefert (Abb. 8), die verschiedene Konstruktionsmerkmale deutlich machen: Die Durchfärbung von Organsystemen zur späteren Identifikation, die treppenförmigen, noch zu schleifenden und zu verspachtelnden Außenflächen sowie verschiedene Bohrungen, um das innerhalb des Modells Hohlräume füllende Wachs der Negativformen ausschmelzen zu können. Auf diese Weise entstanden von diesem Präparat drei verschiedene Modelle für die Ausstellung. Die drei Modelle des 7,5 mm-Embryos sind zwar in Blechschmidts späterer Monographie *Die pränatalen Organsysteme* (1973) vertreten, nicht jedoch in *Der menschliche Embryo* von 1963, weshalb von einer Entstehung innerhalb der Dekade dazwischen auszugehen ist.

## Die Modellsammlung als Werkzeug in Lehre und Vermittlung

**Figure 9:**
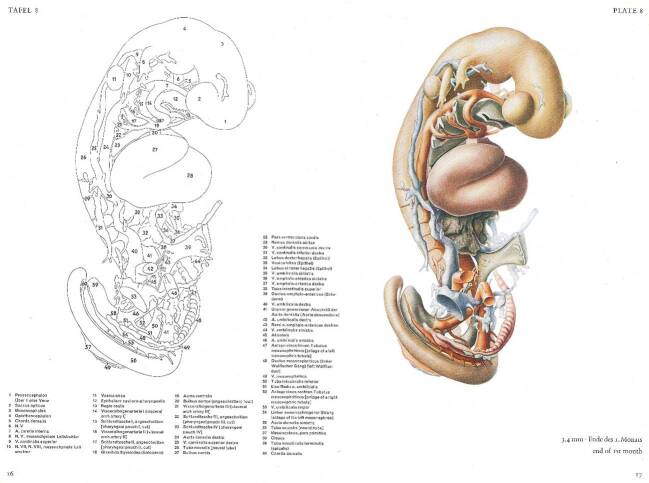

Der Atlas *Der menschliche Embryo* (Blechschmidt 1963a) erschien praktisch zeitgleich zur Aufstellung der Modelle im neu eröffneten Zentrum Anatomie im Jahre 1964 und ist gewissermaßen ein Kommentar zur Modellsammlung als „dreidimensionaler Publikation“ (Ludwig et al. 2014: 11). Auf über 40 Doppeltafeln findet sich jeweils rechts eine detaillierte, farbige Zeichnung eines der Rekonstruktionsmodelle aus dem Ausstellungsraum und links die zugehörige anatomische Legende (Abb. 9). Zwei Jahre zuvor hatte Blechschmidt mit *Die vorgeburtlichen Entwicklungsstadien des Menschen* (1961) auf über 600 Seiten mit fast ebenso vielen Abbildungen, darunter schematische, mikroskopische und Modellzeichnungen sowie Mikrofotografien histologischer Schnitte, embryonaler Körperformen und fetaler Sektionen zu vielen Dutzend seiner Präparate eine umfassende Charakterisierung der menschlichen Embryonalentwicklung vorgelegt. *Der menschliche Embryo* konzentrierte sich nun ganz auf den Ausstellungsraum mit den darin präsentierten Modellen.

Mit der Aufstellung der Modelle in direkter Nachbarschaft des großen Hörsaals der Anatomie in einem öffentlich zugänglichen Ausstellungsraum auf seinerzeit 300 Quadratmetern Fläche und flankiert von einer Begleitpublikation, scheint die Realisierung jener Didaktik abgeschlossen, die Blechschmidt in den 1940er Jahren entwarf. Es fehlen allerdings Quellen um nachzuvollziehen, wie dieses modellbasierte Lehrkonzept in den Vorlesungen umgesetzt wurde und welche Merkmale es im Detail von anderen anatomischen Vermittlungskonzepten abgrenzen. Von einer Wirkung über Göttingen hinaus ist nicht auszugehen, denn weder ist eine vergleichbare Schausammlung bekannt, noch gibt es Hinweise auf eine besondere Rolle der Embryologie in der anatomischen Ausbildung von Mediziner*innen an anderen Standorten auch außerhalb Deutschlands, die auf Blechschmidt zurückgeführt werden könnte.^36^

Die Modelle werden seit der Eröffnung der Ausstellung (vgl. Abb. 1) ohne weiterführende Informationen linear entsprechend der Entwicklungsstufe ausgestellt, wobei die Objektbeschilderung in jeder Vitrine nur Präparatelänge und Entwicklungsalter ausweist. Der fachwissenschaftliche Gehalt lässt sich also nur mit schon vorhandener Expertise, im Rahmen einer Führung oder durch eine begleitende Lektüre erschließen, welche die Entwicklungsprozesse erläutert und so die ausgestellten Zeitschnitte in einen Zusammenhang bringt.

Dass die dekontexualisierte Modellausstellung jedoch nicht notwendig fachwissenschaftlich-embryologisch gelesen werden muss, führt Blechschmidt selbst mit seinem Spätwerk vor: Ab etwa Mitte der 1960er Jahre brachte er sich mit seiner Sammlung verstärkt in den gesellschaftspolitischen Diskurs um Embryonen und Feten sowie insbesondere die Frage nach deren Status in Relation zu geborenen Menschen ein. Spätestens ab 1975 war Blechschmidt als Gründungsmitglied der „Europäischen Ärzteaktion“ institutionalisiert in der sogenannten „Lebensschutzbewegung“ aktiv (Mildenberger 2016a; Ritter 1991b, 1991a), die sich seitdem aus christlich-fundamentalistischer Perspektive für ein generelles Verbot von Schwangerschaftsabbrüchen einsetzt (Sanders et al. 2014; Sanders & Achtelik 2018). Insbesondere vor diesem Hintergrund sollte Blechschmidts 1968 von der Deutschen Verlags-Anstalt herausgegebene, 136 Seiten starke populärwissenschaftliche Darstellung *Vom Ei zum Embryo* gelesen werden, die bis 2008 acht Auflagen erlebte (Blechschmidt 2008).

Sicher nicht ganz zufällig erschien diese Blechschmidt’sche Entwicklungsgeschichte des Embryos ein Jahr nach der deutschen Übersetzung von Lennart Nilssons (1922–2017) 1965 zuerst auf Schwedisch verlegten Werk *Ett barn blir till* (*A Child is Born*). Nilssons farbenprächtige Mikrofotografien lebendig wirkender menschlicher Embryonen in der bildgewaltigen Buchpublikation und einer vorausgegangenen Reportage in der amerikanischen Zeitschrift LIFE prägten maßgeblich und global die visuellen Vorstellungen von ungeborenem menschlichem Leben im späten 20. Jahrhundert und darüber hinaus (Duden 1994; Jülich 2015; Stabile 1997). Möglicherweise auch in Abgrenzung von Nilssons technisch anspruchsvoller fotografischer Inszenierung nutzte Blechschmidt in *Vom Ei zum Embryo* jene stark schematisierende Bildsprache zur Darstellung vorgeburtlicher Entwicklung, die ihm schon in den ersten embryologischen Publikationen eigen war (vgl. insbesondere Blechschmidt 1944, 1948). In diesen Zeichnungen drückt sich sein ausgeprägt mechanizistisches Denken aus, in dem alle Entwicklungsprozesse auf embryonale Wachstumsbewegungen zurückgeführt werden. Blechschmidt erklärt damit auch Verhaltensweisen geborener Menschen und selbst Kulturaspekte wie Zahlsysteme und Körperschmuck zur Konsequenz embryonaler Anlagen:

Der Kragen zum Beispiel verdeutlicht die Halsenge, die nach dem Herabwandern des Herzens in den Brustraum entstanden ist. Ein Kragen kann deshalb als natürlich gelten, weil der Hals angeboren, d. h. entwickelt ist. Eine Halskette symbolisiert die beiden schrägen Halsmuskeln (Musculi sternocleidomastoidei), die seit der zweiten Entwicklungswoche vom Hinterhaupt zum Brustbein ziehen. Eine Halskette, die am Rücken hängen würde, wäre für jedes Lebensalter unrichtig (Blechschmidt 1968: 129).

Während für Nilsson gezeigt werden kann, dass seine Abbildungen gleichermaßen in der liberalen schwedischen Sexualaufklärung als auch von Abtreibungsgegner*innen eingesetzt wurden (Jülich 2017: 306), waren Blechschmidts Bilder Hilfsmittel zur Argumentation eines Personenstatus schon frühester embryonaler Entwicklungsstufen. Verstanden als Beitrag zu einer breiten gesellschaftlichen Debatte, in der Blechschmidt den Embryo einem geborenen Menschen psychosozial und kulturell gleichstellt, wurde sein damit verbundenes Credo „Mensch von Anfang an“ (beispielsweise Blechschmidt 1975, 1986) innerhalb der Lebensschutzbewegung im deutschsprachigen Raum zum geflügelten Wort. Siegfried Ernst (1915–2001) schrieb in 1984 in seiner Grußnote zum 80. Geburtstag Blechschmidts in der von ihm herausgegebenen *Medizin & Ideologie*, der Zeitschrift der Europäischen Ärzteaktion:

Sein Lebenswerk ist eine der Hauptstützen des Kampfes für das Lebensrecht der ungeborenen Kinder auf Weltebene. Millionen von Kindern verdanken ihm deshalb ihr Dasein. Wir sind alle stolz, ihn unseren väterlichen Freund und wissenschaftlichen Leiter nennen zu dürfen und seine Freunde zu sein. (Ernst 1984)

Blechschmidts Rolle in der Lebensschutzbewegung ist bis heute mit der Göttinger Sammlung verbunden, die weiterhin als wissenschaftlicher Beweis für die weltanschauliche Position eines unbegrenzten Schutzes embryonalen menschlichen Lebens verstanden wird. Dies zeigt sich nicht nur auf Webseiten von Lebensschutzorganisationen (etwa o. A. 2019), sondern auch im ausliegenden Gästebuch der Ausstellung, wie an folgendem Eintrag vom 16. Oktober 2016 deutlich wird:

Diese Ausstellung ist so beeindruckend und verdeutlicht, dass das Embryo im 3. Monat schon lange ein Mensch ist und es verboten werden sollte, abtreiben zu dürfen. Es ist spannend zu sehen wie sich das Kind entwickelt und wie vieles schon nach kurzer Zeit fertig ist bzw. so ausgeprägt ist. […] So etwas geniales kann nicht per Zufall entstehen. Gott ist großartig!^37^

Öffentliche Reaktionen wie diese belegen, dass sich in den Modellen nicht nur ein wissenschaftliches Forschungs- und Lehrprogramm materialisiert, sondern damit auch weltanschaulich-ethische Positionen verknüpft werden, die gesellschaftspolitisch wirksam werden können.

## Das Wechselspiel von Individualisierung und Generalisierung im Kontext der Blechschmidt-Sammlung

Wie gezeigt wurde, ist die Blechschmidt-Sammlung als Universitätssammlung in den „Kreislauf von Forschung und Lehre“ eingebunden, zeigt aber zugleich auch Merkmale des Museums als Institution „des Bewahrens, des Deponierens und Exponierens“ (te Heesen 2008: 489). Während die Schnittseriensammlung als Grundlage der Göttinger humanembryologischen Aktivität in der Zeit Blechschmidts im Depot lagert, wurde die Modellsammlung als Ergebnis von Forschung und Instrument der Lehre für die Öffentlichkeit exponiert.

Die histologisch aufbereiteten Embryonen waren dabei Ausgangspunkt und Referenz für unterschiedliche Arten von Visualisierungen, insbesondere für Zeichnungen und Mikrofotografien, nicht zuletzt aber auch die ausgestellten Modelle selbst. Dafür wurden die präparierten Einzelobjekte von ihrer Vorgeschichte als Teil bzw. Gewebe einer Patientin bereinigt und durch zwei neue Informationen charakterisiert: Ihre unter dem Mikroskop gemessene Länge als zentrales Ordnungskriterium und Orientierung über das ungefähre Entwicklungsalter sowie ein Datum. Dieses erlaubt einerseits eine eindeutige Identifikation im Sammlungskontext und markiert andererseits als Präparationsbeginn die Zurichtung im Labor – das Datum der ‚Geburt‘ als Präparat. Dem Reinigungsprozess fielen in der rudimentären Dokumentation sowohl die Einsender*innen als fachlich-soziales Beziehungsgefüge des Sammlers als auch die individuellen Schicksale der Patientinnen zum Opfer. Blechschmidts Kontrolle über die Konstruktion der Göttinger Präparate war dabei total: Mit dem Blick ins Mikroskop entschied er über die Tauglichkeit eines Embryos für die weitere Bearbeitung, wie auch die Auswertung durch ihn in „mikroskopischer ‚Zwiesprache‘ mit den embryonalen Schnittserien […] in stundenlanger Einsamkeit“ (Hinrichsen 1992: 480) erfolgte. Seine Expertise reihte die anonymisierten Präparate, jeweils abgebrochene individuelle Entwicklungsgeschichten, entsprechend ihrer Körperlänge in sein persönliches Archiv einer universellen menschlichen Embryogenese ein.

Mit der Modellierung kehrt die Individualität der Sammlungsdinge zurück, jedoch in neuer Form: Während körperliche (Lehr‑)Modelle etwa in der Botanik üblicherweise Verallgemeinerungen einer Vielzahl von Einzelfällen darstellen und so einen Typus charakterisieren (Olszewski 2011: 290), sind die Rekonstruktionsmodelle aus Wachs oder Kunststoff in der Tradition Borns nach individuellen Schnittserien geformt und damit referenzierte Abbilder konkreter Einzelpräparate. Für den Unterschied zwischen Präparat und Modell hält Hans-Jörg Rheinberger auch mit Blick auf die Anatomie fest:

Das Modell hält sich immer in einem anderen Medium auf, es ist geradezu definiert durch den Übergang vom Gegenstand, den es modelliert, in ein anderes Medium. Während als das Modell immer auch eine Grenze markiert, es also bestenfalls beanspruchen kann, dem Modellierten „täuschend ähnlich“ zu sein, hat das Präparat konstitutiv teil an der Materialität des untersuchten Sachverhalts. (Rheinberger 2006: 3)

Die Humanembryologie als Teilgebiet der Anatomie scheint von diesem Prinzip in einer ähnlichen Weise abzuweichen, wie es jüngst Maria Keil für die Moulagen in der Dermatologie und bemalte Beckenpräparate in der Geburtshilfe diskutiert hat (Keil 2020). Hier wie dort sind Präparat und Modell bzw. Präparateaspekt und Modellaspekt gleichermaßen konstitutiv für die ‚Materialität des Sachverhalts‘. In der Humanembryologie sind die Präparate Schlüsselmomente – zugleich Endpunkt einer Ursprungsgeschichte in Form der dokumentierten medizinischen Gewinnungsprozedur und Ausgangspunkt der Geschichte der Modellierung und Publikation eines wissenschaftlichen Objektes. Wie Morgan anhand des Embryo No. 836 der *Carnegie Collection* gezeigt hat, kann diese zweite Geschichte durch einen Medienwechsel vom körperlichen zum digitalen Modell für manche Präparate bis in die Gegenwart führen: „836 and its embryo colleagues are more active than ever, somersaulting for the cameras, traveling through cyberspace, rehearsing for their television debut, and showing that even dead embryos can have long and productive lives“ (Morgan 2004: 7).

Eine solche Produktivität benötigt jedoch weder eine Verlagerung in den virtuellen Raum, noch eine extensive Verdatung, wie die Blechschmidt-Sammlung zeigt. Ganz im Gegenteil ist hier die Modellsammlung geradezu raumumfassend körperlich und völlig von ihrem Entstehungsprozess entkoppelt: Im Ausstellungsraum werden die Modelle seit den 1960er Jahren unkommentiert präsentiert. Der Ausstellungstext beschränkt sich auf Beschriftungsschilder in den einzelnen Modellvitrinen, die die Körperlänge und das ungefähre Entwicklungsalter in Wochen angeben. Es bleibt damit sogar offen, auf welche konkrete Schnittserie sich ein jeweiliges Modell bezieht.

Zweifellos bestehen zwar große Ähnlichkeiten zwischen der Göttinger Sammlung und anderen Referenzsammlungen wie der des *Carnegie Department of Embryology* bezogen auf die Sammelstrategien, den Netzwerkaufbau, die konkreten Gewinnungsprozesse und nicht zuletzt die Umwandlung der histologischen Schnittserien in Forschungsobjekte durch die Rekonstruktionsmodellierung. In der massiven Verschiebung des Fokus von der Präparate- auf die Modellsammlung ausgehend von einem eigenwilligen Lehrkonzept ist die Unternehmung Blechschmidts hingegen einmalig. Voller sinnstiftender wissenschaftlicher Objekte, jedoch ohne eine im Raum installierte Vermittlungsebene, ist der sichtbare Teil der Blechschmidt-Sammlung im Sinne von Anke te Heesen und Margarete Vöhringer ein Paradebeispiel für jene wissenschaftlich-musealen „Objektakkumulationen [die] sich den gängigen Beschreibungen von Museum, Archiv, Sammlung oder Ausstellung entziehen und vielmehr auf die Bedeutung des Präsentierens und Darstellens in wissenschaftlichen Räumen verweisen.“ (te Heesen & Vöhringer 2014: 9)

Der Präsentationsmodus der Modellsammlung erlaubt es dabei, die nach individuellen Schnittserien geformten und nur von Expert*innen interpretierbaren Modelle einzelner Entwicklungsstufen zugleich als räumlich organisiertes Modell der menschlichen Embryonalentwicklung zu lesen. Zentrale Aussagen der Humanembryologie lassen sich dem Embryologen und Kustos der Blechschmidt-Sammlung Jörg Männer zufolge,

[…] nicht an einzelnen Repliken festmachen, die lediglich statische Momentaufnahmen eines dynamischen Prozesses repräsentieren, sondern erfordern eine Gesamtschau der Embryogenese. Die Blechschmidt-Sammlung ermöglicht eine solche Gesamtschau und fungiert in ihrer Gesamtheit als ein Modell der Embryonalentwicklung […]. (Männer 2014: 36)

Diese fachwissenschaftliche Perspektive wird allerdings durch eine zweite überlagert, die Blechschmidt mit der öffentlichen Zugänglichkeit als quasi-museale Ausstellung angelegt hat. Blechschmidt griff dafür mit seiner populärwissenschaftlich-weltanschaulichen Deutung (etwa Blechschmidt 1974, 1979, 1982) auf den fachwissenschaftlich-embryologischen Ausstellungsraum zu, obgleich er ab 1974 nicht mehr Institutsleiter war und bald darauf Göttingen verließ, um sich in Freiburg im Breisgau niederzulassen.

Sammlungen wie diese gehen damit nicht notwendig in jenem „Kreislauf von Forschung und Lehre“ auf, dem sie entstammen, sondern zeichnen sich durch eine eigentümliche „semi-autonomy“ (Jardine et al. 2019: 4) aus, die sie von institutionellen Rahmenbedingungen und disziplinären Trends entkoppeln kann. So können selbst humanembryologische Rekonstruktionsmodelle für eine weltanschauliche Debatte instrumentalisiert werden, für die sie eigentlich ungeeignet sind. Wie Hopwood an einer marmornen Portraitbüste von His gezeigt hat, der das Modell eines Embryos vom Ende der vierten Entwicklungswoche hält, ist das Modellierte zu signifikant und zu sehr an disziplinäre Praktiken gebunden, um als Symbol insbesondere in der Abtreibungsdebatte verwendet zu werden:

Too early for a layperson to recognize as human, the model represents a particular preparation, not a generalized icon. [T]he disturbing effect—is that man really holding an embryo?—thematizes more than it mystifies the labour through which human embryology made its objects at a scale that can be held in the hand. (Hopwood 2012: 25–26)

Der Umstand allerdings, dass es sich um bestimmte, individuelle Präparate handelt, ist im Falle Blechschmidts nicht nur in der Ausstellung der Modellsammlung unsichtbar, sondern auch in dessen populärwissenschaftlichen Texten. In *Vom Ei zum Embryo* endet das einführende Kapitel „Wozu Embryologie?“ mit einer knappen Darstellung des Göttinger Sammlungskontextes. Blechschmidt versteht die Modellsammlung darin als „Dokumentationsmaterial“, dem „über 200.000 mikroskopische Einzelpräparate besonders ausgesuchter Frühstadien zugrunde[liegen].“ (Blechschmidt 1968: 20) Bezogen auf die Anzahl der Objektträger ist dies der Gesamtumfang der über bis dahin über 24 Jahre aufgebauten Schnittseriensammlung, die mit irritierender Bescheidenheit auf einen Halbsatz reduziert wird. Im direkten Anschluss gibt Blechschmidt den technischen Aufwand der Modellproduktion mit „mehr als eineinhalb Jahrhunderten Arbeitsstunden“ (ebd.: 20) an, der Ressourcenbedarf für den Aufbau der Schnittseriensammlung hingegen bleibt unerwähnt.

Diese Unsichtbarkeit der Hopwood zufolge eigentlich signifikanten Präparate hängt weniger mit einer breiten Öffentlichkeit als Zielgruppe, sondern vielmehr Blechschmidts Interessen in Forschung und Lehre zusammen. Dieser gibt auch in Fachpublikationen (etwa Blechschmidt 1947, 1955, 1963b, 1967) regelmäßig nicht genügend Informationen zu Präparaten, um sie in der Sammlung identifizieren und damit als individuell bestimmen zu können. Sein „Dokumentationsmaterial“ soll weniger Qualitäten, als vielmehr Quantitäten verkörpern: Drückende und ziehende Zellmassen, deren Wachstum, Wanderung und Schrumpfung Organbildung erst auslöst und dann darstellt. Als Entwicklungsreihe präsentiert sollen die einzelnen Zeitschnitte nicht jeweils als individuell menschlich erkannt werden, sondern sind menschlich in ihrer körperlichen Kontinuität: „Der Mensch wird nicht Mensch, sondern ist ein Mensch, und zwar in jeder Phase seiner Entwicklung.“ (Blechschmidt 1968: 32) Präsentiert als Gegenüber in Augenhöhe und mit vergleichbarer Torsolänge werden modellierte Embryonen und erwachsene Betrachter*innen von Blechschmidt physisch gleichgestellt. Obgleich konkret und individuell wie alle embryologischen Rekonstruktionsmodelle konnten die von Blechschmidt in ihrer schaugestellten Kontinuität dadurch zu generalisierten Ikonen der Antiabtreibungsbewegung in Deutschland werden.

## Danksagung

Unterstützt wurde das Projekt in finanzieller wie ideeller Hinsicht umfassend durch die Zentrale Kustodie der Universität Göttingen unter Leitung von Dr. Marie Luisa Allemeyer sowie die Universitätsmedizin Göttingen und insbesondere die Abteilung Anatomie und Embryologie am Zentrum Anatomie unter Leitung von Prof. Dr. Christoph Viebahn. Ich danke Prof. Dr. Claudia Wiesemann für die Möglichkeit, im Wintersemester 2019/20 am Institut für Ethik und Geschichte der Medizin der Universitätsmedizin Göttingen das Artikelmanuskript zu erstellen. Besonderer Dank gebührt darüber hinaus Gutachter*in B, welche*r mit umfangreichen und detaillierten Anmerkungen und Hinweisen erheblich zur finalen Fassung beigetragen hat.

## Footnotes

1 Die Blechschmidt-Sammlung besteht aus einem Modellteil mit 61 Rekonstruktionsmodellen in 60 Vitrinen sowie einem Präparateteil mit 430 histologischen Schnittserien menschlicher Embryonen und Feten. Die gemeinsam mit den Schnittserien gelagerte Dokumentation der Sammlung umfasst neben Laborprotokollen zur Einbettung von mehreren Hundert Präparaten vorrangig im Zeitraum 1944 bis 1961 auch farbig kodierte Umzeichnungen ganzer Schnittserien. Zusätzlich finden sich in der Institutsbibliothek mehr als 100 Sonderdrucke Blechschmidts und etwa 40 von ihm betreute Doktorarbeiten. Im Rahmen des Provenienzforschungsprojektes wurden dieser Dokumentation verschiedene Bestände hinzugefügt, die wie die übrige Sammlungsdokumentation bei berechtigtem Forschungsinteresse auf Anfrage am Zentrum Anatomie, Abteilung Anatomie und Embryologie (http://www.embryologie.uni-goettingen.de), zugänglich sind. Diese neuen Bestände umfassen 8.325 Glasnegative und -dias, die vorrangig Präparate, Zeichnungen und Modelle zeigen; 106 Einbettungs- und mehrere Hundert Makropräparate aus verschiedenen Forschungs- und Lehrkontexten sowie einen umfangreichen Schriftwechsel insbesondere zu 504 Einlieferungsvorgängen von Embryonen und Feten von 1943 bis 1969, zu Publikationen Blechschmidts und studentischen Angelegenheiten. Der Schriftwechsel wurde durch den Verfasser zum Zwecke der Bestandssicherung aus Ordnern oder Bündeln loser Blätter entsprechend der ursprünglichen Sortierung in Archivkartons umgelagert und wird in den folgenden Fußnoten mit den dabei vergebenen Inventarnummern als „Archiv Blechschmidt“ zitiert.

2 Die Ersterwähnung des Verfahrens ohne hinreichende Beschreibung für eine Reproduktion desselben erfolgte schon einige Jahre zuvor (Born 1877).

3 Alle Daten: UAG, Kuratorium. Medizinische Fakultät. Prof. Dr. Blechschmidt XVI. III a.b.17, Bd. 5 [Personalblatt].

4 UAG, Kuratorium. Medizinische Fakultät. Prof. Dr. Blechschmidt XVI. III a.b.17, Bd. 1, Bl. 108.

5 UAG, Kuratorium. Medizinische Fakultät. Prof. Dr. Blechschmidt XVI. III a.b.17, Bd. 7 [ohne Blattnummern], Blechschmidt an Niedersächsischen Kultusminister, 24.07.1972, Niedersächsischer Kultusminister an Blechschmidt, 12.03.1973 sowie 03.11.1973.

6 Archiv der Anatomie der Universität Prag, Box 3, „Handbuch“, Hochstetter an Grosser, 09.02.1943.

7 Universitätsbibliothek Würzburg, Nachlass Elze, Mappe 16, Elze an Romeis, München, 27.06.1946.

8 Zentrum Anatomie, Archiv Blechschmidt, B4, Blechschmidt an Forschungsgemeinschaft der Deutschen Wissenschaft, Berlin, 29.06.1942

9 Bundesarchiv, DS, R73/10341, Blechschmidt an Präsidenten des Reichsforschungsrats, Berlin, 22.06.1944, [Bl. 2]

10 Zentrum Anatomie, Archiv Blechschmidt, B4, Blechschmidt an Guirao, Instituto Anatomico, Universität Granada, 24.10.1951.

11 Zentrum Anatomie, Archiv Blechschmidt, E1, Belonschkin, Posen, an Blechschmidt, 13.02.1943.

12 Zentrum Anatomie, Archiv Blechschmidt, B5, Blechschmidt an Martius, 13.12.1943.

13 Zentrum Anatomie, Archiv Blechschmidt, E1, Direktor der Landesfrauenklinik, Celle, an Blechschmidt, 12.05.1943 sowie Blechschmidt an Landesfrauenklinik Celle, 17.07.1943.

14 Zentrum Anatomie, Archiv Blechschmidt, E1, Blechschmidt an Landesfrauenklinik Celle, Hannover, […], 20.01.1947.

15 Zentrum Anatomie, Archiv Blechschmidt, E3, A., Salzgitter, an Blechschmidt, 19.12.1962. Blechschmidt an C., Oldenburg, 13.12.1962.

16 Zentrum Anatomie, Archiv Blechschmidt, E2, S., Schwarmstedt, an Blechschmidt, 18.11.1947, Zentrum Anatomie, Archiv Blechschmidt, E3, E., Celle, an Blechschmidt, 27.03.1958.

17 Zentrum Anatomie, Archiv Blechschmidt, E3, Blechschmidt an G., Leer, 05.06.1950.

18 Zentrum Anatomie, Archiv Blechschmidt, E2, Blechschmidt an B., Verden, 07.10.1949.

19 Zentrum Anatomie, Archiv Blechschmidt, E1, Blechschmidt an Belonoschkin, Posen, 12.04.1944.

20 Zentrum Anatomie, Archiv Blechschmidt, E2, Mitarbeiter des Instituts an S., Salzwedel, 12.08.1958.

21 Aus diesem Jahr stammt der letzte überlieferte Brief zu einer Einsendung und das letzte Präparat in der Schnittseriensammlung, auch wenn Blechschmidt zumindest bis zu seiner Emeritierung 1973 weiter Präparate erhält.

22 Vgl. Zentrum Anatomie, Archiv Blechschmidt, Blechschmidt Manuskripte […], Junges Ei 1952 [Typoskript], unveröffentlicht, S. 1.

23 Zentrum Anatomie, Archiv Blechschmidt, E2, Blechschmidt an B., Freiburg, 31.05.1949.

24 Mutmaßlich sind vor 1945 auch Präparate aus eugenischen Zwangsabbrüchen in die Sammlung eingegangen, die an den zwei nachweislich liefernden Kliniken in Göttingen und Bad Lauterberg durchgeführt wurden (Koch 1994: 24, 44, 65).

25 Zentrum Anatomie, Archiv Blechschmidt, E2, Blechschmidt an F., Saarbrücken, 17.07.1950; auch E2, Blechschmidt an H., Freiburg, 25.01.1952.

26 Zentrum Anatomie, Archiv Blechschmidt, E1, D., Holzminden, an Blechschmidt, 25.04.1949.

27 Zentrum Anatomie, Archiv Blechschmidt, E3, A., Salzgitter, an Blechschmidt, 17.01.1965.

28 Zentrum Anatomie, Archiv Blechschmidt, B2, Blechschmidt an Fa. Paul Kienzle, Apparate und Modellbau, Stuttgart, Bad Cannstatt, 21.12.1942. Aktenbündel II, Pauwels, Aachen, an Blechschmidt, 30.11.1946.

29 Interview mit Michael Brandt am 8. August 2017.

30 Es handelt sich dabei um ein 2007 aus Styroporplatten konstruiertes Modell.

31 UAG, Kur. 0985 Etat und a. o. Bewilligungen. XVII III C a 4 IV v. 01.01.1948- [58] Blechschmidt an Kuratorium, 21.05.1953

32 Telefongespräch mit Egbert von Bischoffshausen am 19. Februar 2019.

33 (Kühn 1950: 1). Der Vermerk einer Herkunft aus Spontanabort findet sich auch auf der entsprechenden Karteikarte zum Präparat in einem älteren Inventar, der „Embryonenkartei“ (Archiv Sammlung Blechschmidt).

34 Zentrum Anatomie, Archiv Blechschmidt, E2, Blechschmidt an P., Barnstorf, 02.11.1945.

35 Über die technische Assistentin ist außer dem Namen wenig bekannt. Die Tätigkeitsdauer wurde aus der Handschrift in den Einbettungsprotokollen abgeleitet.

36 Unbenommen bleibt natürlich, dass Embryologie generell Teil medizinischer Curricula und (virtuelle) Modellierung als Teil der Lernumgebung auch weiterhin relevant ist (Chekrouni et al. 2020).

37 Gästebuch der Blechschmidt-Ausstellung, 1997–2020, unpaginiert, 16.10.2016.
